# Efficacy and Safety of Acupuncture at Sensitized Acupoints for Knee Osteoarthritis: Protocol for a Multicenter, Single-Blind Randomized Controlled Trial

**DOI:** 10.2196/77336

**Published:** 2025-09-24

**Authors:** Zenan Wu, Zehao Hu, Zixuan Xu, Tao Xiao, Qiuxuan Huang, Xu Zhou, Haifeng Zhang, Yong Fu

**Affiliations:** 1 Clinical Medical College Jiangxi University of Chinese Medicine Nanchang City China; 2 Shantou Hospital of Traditional Chinese Medicine Guangzhou University of Traditional Chinese Medicine Shantou China; 3 Evidence-based Medicine Research Center Jiangxi University of Chinese Medicine Nanchang City China; 4 Department of Acupuncture and Moxibustion The Affiliated Hospital of Jiangxi University of Chinese Medicine Nanchang City, Jiangxi Province China

**Keywords:** randomized controlled trial, knee osteoarthritis, acupuncture, acupoint sensitization, efficacy, safety

## Abstract

**Background:**

Knee osteoarthritis (KOA) is a prevalent osteoarthritic disorder. Although acupuncture is increasingly used in clinical practice for KOA management, its efficacy remains to be further optimized.

**Objective:**

This trial aims to evaluate the efficacy and safety of acupuncture at sensitized acupoints for the treatment of KOA.

**Methods:**

We will recruit 350 patients diagnosed with KOA from 3 clinical centers in this single-blind, sham-controlled, randomized controlled trial. Participants will be randomized to receive either acupuncture at 5 high-probability sensitized acupoints or sham acupuncture with nonpenetrative needling using Takakura acupuncture simulation devices. Both groups will receive 24 sessions over 8 weeks, followed by a 16-week posttreatment follow-up period. The primary outcome is the proportion of responders, defined as a reduction of 2 or more points in the Numeric Rating Scale score at week 8. Secondary outcomes include changes in scores on validated scales for KOA severity, walking distance, disability, depression, anxiety, insomnia, and pain self-efficacy. Adverse events will be documented for safety evaluation.

**Results:**

The first participant was enrolled on February 15, 2025, and by June 28, 2025, we had enrolled 25 patients. Data analysis has not yet been initiated. The completion of data collection is anticipated by March 2026.

**Conclusions:**

This trial aims to provide confirmatory and exploratory evidence regarding the efficacy and safety of sensitized acupoint-based acupuncture for treating KOA. If the hypothesized benefits are substantiated, sensitized acupoint-based acupuncture could emerge as a complementary and alternative therapy for KOA, potentially reducing reliance on medication and mitigating drug-related adverse effects.

**Trial Registration:**

ClinicalTrials.gov NCT06805188; https://clinicaltrials.gov/study/NCT06805188

**International Registered Report Identifier (IRRID):**

DERR1-10.2196/77336

## Introduction

### Background

Knee osteoarthritis (KOA), which is a prevalent chronic degenerative joint disorder, severely impairs the functional capacity and quality of life of affected individuals. As a primary cause of disability among middle-aged and older adult populations, the incidence of KOA has risen significantly because of global demographic aging trends [1,2]. Epidemiological studies indicate that approximately 40% of individuals aged 50 years or older exhibit symptomatic KOA, with the prevalence increasing to 60% among those aged 65 years or older [3]. Notably, the increasing prevalence of obesity and sports-related injuries has contributed to a growing incidence of KOA in younger cohorts [4]. This trend is reflected in the rising use rate of total knee arthroplasty (TKA) among younger patients, whose proportion among all TKA procedures increased from 38.4% (2001-2005) to 42.7% (2006-2010) [5]. In addition to causing persistent pain and functional impairment, KOA imposes substantial socioeconomic burdens, with annual direct and indirect health care expenditures reaching US $136 billion in the United States [6] and US $12.1 billion in China [7]. Moreover, KOA is frequently associated with comorbidities, such as depression, anxiety, and cardiovascular diseases, further compromising patients’ quality of life [8,9].

Current guideline-recommended management strategies for KOA primarily include pharmacological interventions, exercise therapy, and surgical options [10], all of which are associated with inherent limitations and adverse effects. Pharmacotherapy, as the most frequently used conservative approach, includes nonsteroidal anti-inflammatory drugs, analgesics, and corticosteroids. Although these agents provide short-term relief from pain and inflammation, their prolonged use elevates the risk of adverse reactions, such as gastrointestinal hemorrhage, renal impairment, and cardiovascular events [11,12]. A large-scale meta-analysis demonstrated that long-term nonsteroidal anti-inflammatory drug administration increases the risk of cardiovascular events by 20% to 50% [11]. Opioid use in KOA management remains restricted in international guidelines because of addiction potential and severe side effects [13,14]. With respect to exercise therapy, Cochrane systematic reviews indicate that while exercise interventions achieve short-term improvements in pain and functional capacity among patients with KOA, their long-term efficacy remains inconclusive [15,16]. Suboptimal adherence also substantially compromises therapeutic outcomes; studies report that 40% to 50% of patients with KOA discontinue long-term exercise regimens [17]. For advanced KOA, surgical interventions, such as TKA, may be indicated. However, perioperative risks and substantial postoperative care costs limit accessibility [18]. Approximately 20% of patients who have TKA experience persistent postoperative pain [19]. In addition, finite prosthesis longevity necessitates potential revision surgeries for younger patients, thereby increasing complication risks [20].

Given the limitations of routine therapies, identifying safe and effective alternative or adjunctive interventions for KOA has become imperative. Acupuncture, a core therapeutic modality within traditional Chinese medicine, has been used for centuries in pain management with broad clinical applications [21]. The proposed mechanisms underlying acupuncture analgesia include promoting local circulation, reducing inflammatory responses, modulating neuroendocrine function, and activating endogenous pain-inhibitory pathways [22,23]. Preclinical studies suggest that acupuncture may alleviate articular inflammation by downregulating proinflammatory cytokines, such as tumor necrosis factor-α and interleukin-1β [24]. Neurophysiological investigations have indicated that acupuncture mediates analgesia through central modulation of pain pathways, particularly by activating descending inhibitory systems [25]. Furthermore, emerging evidence suggests that acupuncture may influence KOA progression through regulation of gut microbiota, suggesting novel directions for mechanistic research [26].

Although several randomized controlled trials (RCTs) have explored the efficacy of acupuncture for KOA [25,27-29], their limitations preclude definitive conclusions. For instance, they used shallow needling as a sham acupuncture control, despite evidence that shallow needling may produce substantial therapeutic effects [30]. Additional limitations include predominantly single-center designs with insufficient sample sizes, unvalidated blinding results, and short follow-up periods that fail to capture sustained treatment benefits. To address these gaps, we propose a rigorously designed, large-scale, multicenter RCT to evaluate acupuncture’s efficacy in KOA. This trial will use the validated nonpenetrating Takakura sham acupuncture device to minimize placebo effects and apply the James and Bang indices to assess blinding success. We will use validated assessment tools to systematically evaluate the multidimensional effects of acupuncture on pain, physical function, and quality of life in patients with KOA [31,32]. In addition, we will focus on the sustained effects after treatment cessation through a 24-week extended follow-up and document safety profiles to comprehensively determine the clinical utility of this sensitization-guided acupuncture approach.

Furthermore, previous RCTs exhibited heterogeneity and inadequate justification in acupoint selection. In clinical practice, we have found that there is potential for optimizing acupoint prescriptions in KOA management through the phenomenon of acupoint sensitization. Acupoint sensitization is defined as enhanced responsiveness of specific acupoints to mechanical stimuli (eg, acupuncture and acupressure) under pathological conditions. Applying acupuncture to sensitized acupoints facilitates the induction of “kuai ran” (translated as “comfortable sensation”) and “de qi” responses (eg, soreness, numbness, distension, or heaviness). Theoretically, this approach enhances therapeutic efficacy by regulating qi blood flow in meridians, improving periarticular circulation, reducing inflammatory responses, and alleviating pain and swelling. Expert consensus [33] and cross-sectional evidence [34] further emphasize that sensitization status is a critical characteristic for acupoint selection in KOA. Therefore, to address heterogeneity in acupoint selection and optimize therapeutic outcomes, we propose targeting the most frequently sensitized acupoints. Through a multimodal approach combining pain threshold measurement, infrared thermography, and data mining analysis, we identified 5 acupoints exhibiting the highest mechanical sensitization frequency: Chize (LU5), Quchi (LI11), Dubi (ST35), Fengshi (GB31), and Xiyangguan (GB33) [33,35].

### Objective

This RCT aims to generate confirmatory evidence for the efficacy of acupuncture based on sensitization-guided acupoint selection in KOA, implemented through standardized treatment protocols.

## Methods

### Ethical Considerations

This research protocol was approved by the ethics committee of the First Affiliated Hospital of Jiangxi University of Traditional Chinese Medicine (JZFYLL20250103002) and was prospectively registered on ClinicalTrials.gov (NCT06805188). The informed consent form will be explained by the investigator to participants in clear and understandable language, covering the interventions, associated risks, potential benefits, and the participant’s rights within the trial. The form will be signed by the participant. If a participant wishes to withdraw from the trial, final analysis will be conducted using the available data, and their wishes will be accommodated accordingly. Participant confidentiality will be ensured, and all data will be deidentified in compliance with ethical standards.

### Study Design

**Figure 1:**
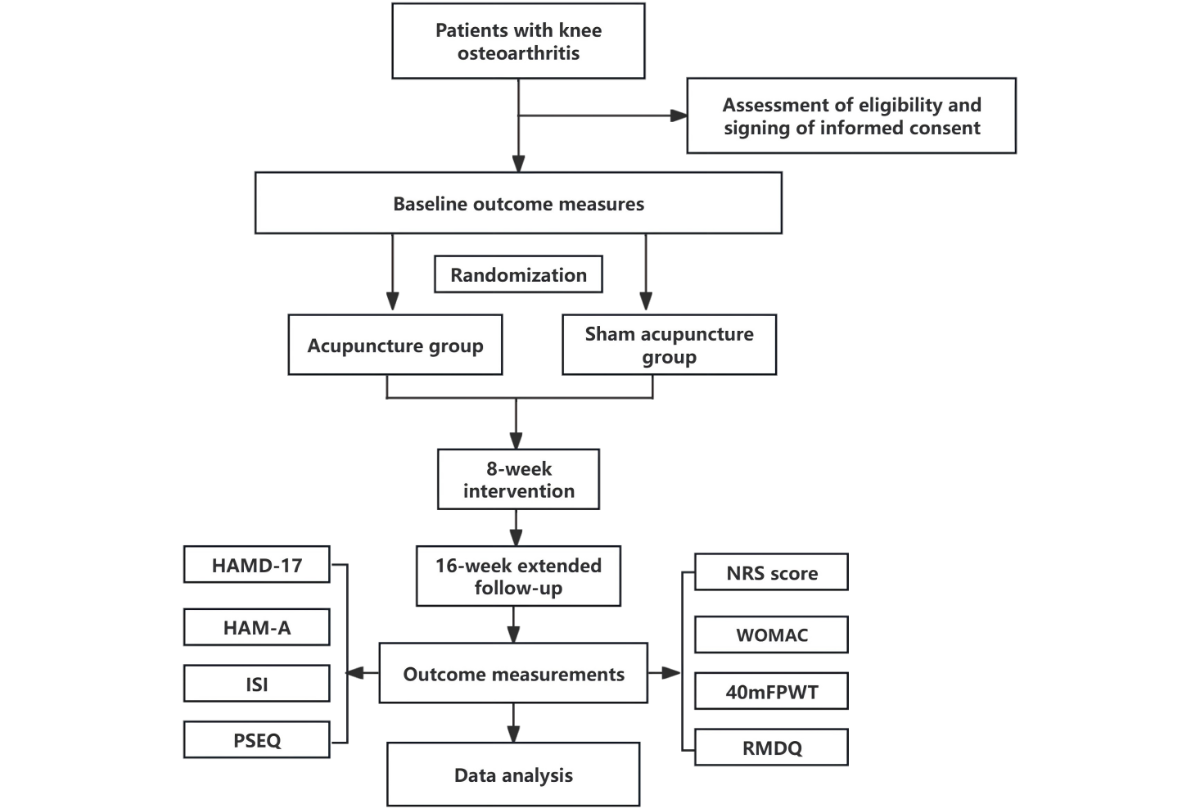
The study procedure. This schematic illustrates the structured flow of a randomized controlled trial designed to assess the therapeutic effects of acupuncture compared to sham acupuncture in patients diagnosed with knee osteoarthritis. 40mFPWT: 40 m Fast-Paced Walk Test; HAM-A: Hamilton Anxiety Rating Scale; HAMD-17: Hamilton Depression Rating Scale-17; ISI: Insomnia Severity Index; NRS: Numerical Rating Scale; PSEQ: Pain Self-Efficacy Questionnaire; RMDQ: Roland-Morris Disability Questionnaire; WOMAC: Western Ontario and McMaster Universities Osteoarthritis Index.

This study is a multicenter, single-blind, sham-controlled RCT. The study protocol was developed in compliance with the SPIRIT (Standard Protocol Items: Recommendations for Interventional Trials) guidelines (Multimedia Appendix 1) [36]. The study procedure is presented in Figure 1.

### Study Sites and Recruitment

Participants will be recruited from the Affiliated Hospital of Jiangxi University of Traditional Chinese Medicine, the Second Affiliated Hospital of Jiangxi University of Traditional Chinese Medicine, and the Yuxi Hospital of Traditional Chinese Medicine. Recruitment notices will be posted on hospital bulletin boards, social media platforms, and official WeChat accounts of participating institutions. Potential participants expressing interest will receive an informed consent form outlining the study protocol, potential risks and benefits, and participant rights. Upon confirmation of voluntary participation, eligible individuals will undergo screening assessments to verify eligibility. Formal enrollment will commence only after written informed consent is obtained.

### Eligibility Criteria

The inclusion and exclusion criteria for the patients are presented in Textbox 1.

**Inclusion and exclusion criteria.**

**Inclusion criteria**

- A diagnosis of knee osteoarthritis (KOA) according to the American College of Rheumatology criteria [37], defined by at least 1 of the following combinations: knee pain on most days during the preceding month and radiographic osteophyte formation; knee pain and synovial fluid analysis consistent with osteoarthritis, morning stiffness for 30 minutes or less, and crepitus ; knee pain and being aged 40 years or older, morning stiffness for 30 minutes or less, and crepitus
- Aged between 18 and 70 years, accounting for the increasing prevalence of KOA in younger populations to enhance generalizability
- Persistent knee pain for 3 or more months with a pain intensity of 4 or more points on the Numerical Rating Scale at screening
- Provision of written informed consent

**Exclusion criteria**

- History of previous knee surgery
- Treatment with KOA-targeted therapies within the preceding 24 weeks, including intra-articular corticosteroid injections, acupuncture, or moxibustion (this criterion aims to minimize residual effects from previous therapies, as acupuncture efficacy may persist for 12-20 weeks after treatment [38])
- Comorbidities potentially confounding knee pain assessment, such as fractures, synovial cysts, or rheumatoid arthritis
- Severe degenerative disorders or neurological impairments (eg, stroke and Guillain-Barré syndrome) causing knee disability
- Documented history of severe psychiatric disorders, organ failure, or malignancy
- Scheduled knee surgery within the next 3 months
- Pregnancy or lactation
- Concurrent participation in other clinical trials

### Randomization and Allocation Concealment

Eligible participants will be randomly assigned to either the acupuncture group or the sham acupuncture group. The randomization sequence will be created by an independent center not involved in study objectives, participant recruitment, and follow-up procedures, using the PLAN procedure (SAS Institute Inc) with variable block sizes of 4 and 6. The resulting allocation sequence will be concealed in sequentially numbered, sealed, and opaque envelopes. After eligibility confirmation, the assigned acupuncturist will sequentially access the corresponding envelope to receive the group assignment.

### Blinding Procedures

Study participants, outcome assessors, and data analysts will remain blinded throughout the trial. The blinding of acupuncturists will not be maintained due to the inherent nature of the interventions. To minimize bias risks from unblinded acupuncturists, we will implement multiple measures. First, a rigorous standardized operating procedure has been established (Multimedia Appendix 2). All treatments administered by acupuncturists must adhere to predefined acupuncture parameters (including point location, needle retention time, stimulation techniques, etc), with compliance monitored on-site by clinical research coordinators. Second, nontherapeutic discussions between acupuncturists and participants will be limited; for example, conversations regarding treatment efficacy expectations or additional lifestyle advice are prohibited to reduce interference from the acupuncturists’ unblinded status. Unblinding will be strictly limited to instances of serious adverse events (AEs) requiring immediate clinical management.

### Interventions

#### Acupuncture Group

**Figure 2:**
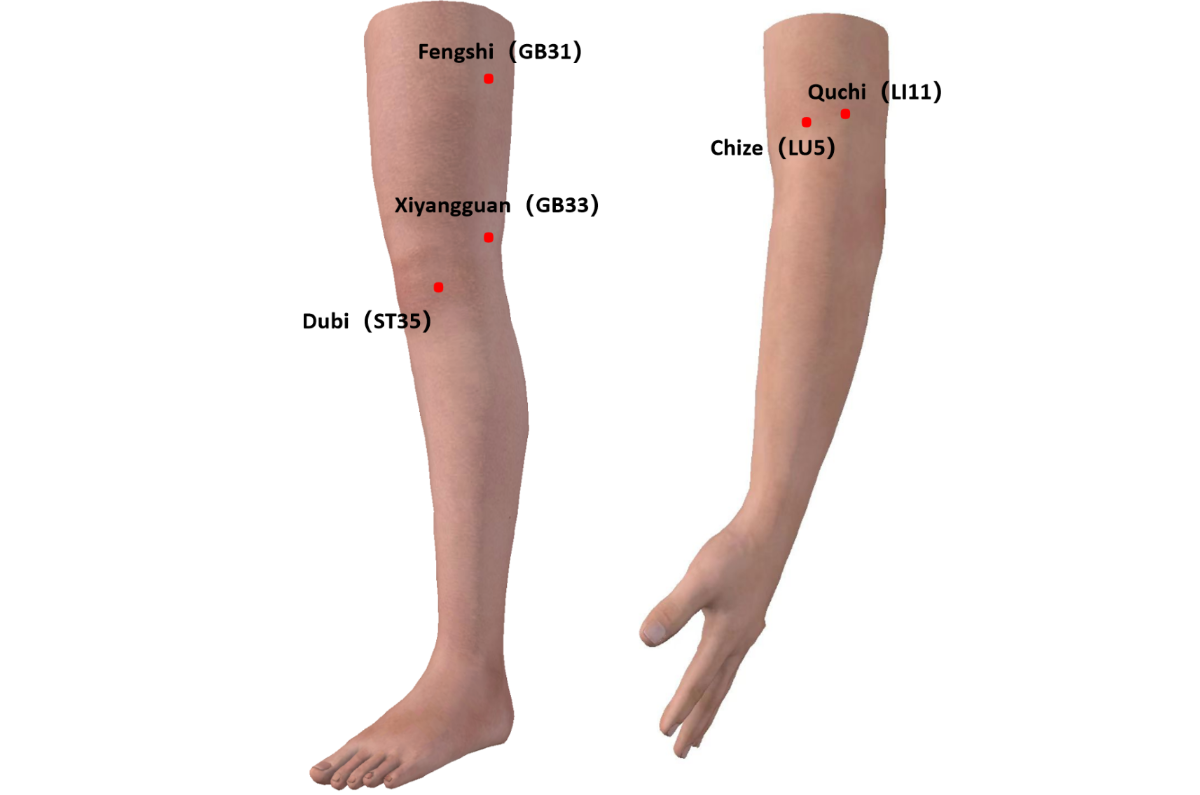
Anatomical location of selected acupoints. Diagram used with permission from www.3Dbody.com [42].

Participants assigned to the acupuncture group will receive 24 sessions administered three times per week over 8 weeks. Treatments will be performed by licensed acupuncturists who have more than 10 years of clinical experience and have also undergone a standardized 2-week protocol-specific training program before trial commencement. On the basis of previous measurements of tenderness thresholds and temperature sensitivity, 5 acupoints with the highest propensity for sensitization to acupuncture stimulation (mechanical pressure pain threshold of ≥2372 gf; thermal threshold of ≥32°C) will be selected for the acupuncture prescription [34,39,40]. These include Chize (LU5), Quchi (LI11), Dubi (ST35), Fengshi (GB31), and Xiyangguan (GB33) on the affected side. Acupoint localization will adhere to the *World Health Organization Standard Acupuncture Point Location in the Western Pacific Region* [41] (Figure 2 and Table 1 [42]). Sterile disposable acupuncture needles (Huatuo brand; 0.30 mm diameter×40 mm length) will be used. Needle insertion will be followed by manual stimulation to elicit “de qi” sensations (characterized by soreness, numbness, distension, or heaviness). Each acupoint will receive approximately 30 seconds of manipulation, with the intensity calibrated to individual tolerance thresholds. Needles will be retained for 30 minutes per session, supplemented by additional manual stimulation at 10-minute intervals.

**Table 1:** Anatomical location of selected acupoints.

Acupoint	Location
LU5 (Chize)	On the anterior cubital crease, radial to the tendon of the biceps brachii muscle
LI11 (Quchi)	On the lateral aspect of the elbow, at the midpoint of the line connecting the lateral end of the cubital crease and the lateral epicondyle of the humerus, measured with the elbow in flexion
ST35 (Dubi)	On the anterolateral aspect of the knee, within the lateral depression of the patellar ligament
GB31 (Fengshi)	On the lateral midline of the thigh, 20 cm proximal to the popliteal crease, in the depression midway between the greater trochanter and the lateral femoral epicondyle, which lies between the vastus lateralis and the biceps femoris muscles
GB33 (Xiyangguan)	On the lateral aspect of the knee, in the depression immediately superior to the lateral epicondyle of the femur

#### Sham Acupuncture Group

**Figure 3:**
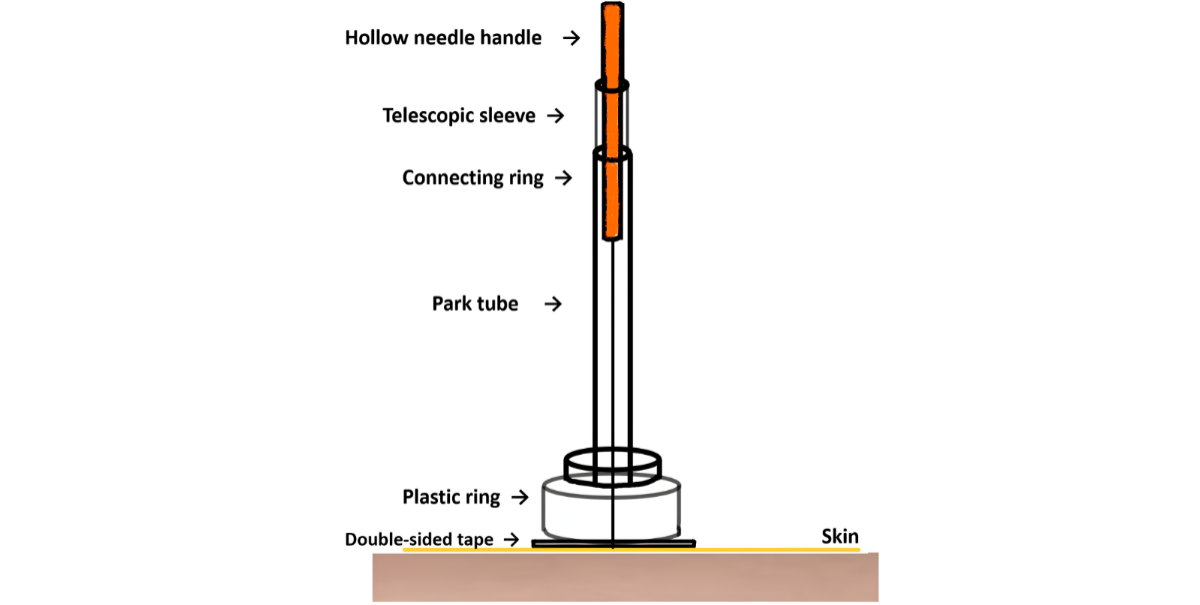
Sham acupuncture device.

The participants assigned to the sham acupuncture group will receive nonpenetrating stimulation at identical acupoint locations to those in the acupuncture group, which will be administered using validated Takakura nonpenetrating sham devices (Figure 3). The construction of this device includes a hollow needle handle, a retractable sleeve, a connecting ring, a stabilizing Park tube (telescope structure), a plastic adhesive ring, and double-sided tape. The device is affixed to the skin surface via double-sided tape, forming a stable platform. The retractable sleeve mechanism within the hollow needle handle creates tactile feedback, mimicking needle insertion while preventing actual skin penetration. The Park tube maintains structural integrity, preventing accidental dislodgement or penetration. Specifically designed blunted-tip needles, visually indistinguishable from verum acupuncture needles, are deployed. During application, the sham needle tip contacts the skin surface without penetration. A standardized 30-second simulated manipulation procedure, replicating the manual technique for the acupuncture group, is performed, ensuring equivalent sensory experience despite the absence of skin penetration. All treatment parameters, including acupoint selection (Figure 2 [42]), needle retention time (30 min), treatment frequency (thrice weekly), treatment course (8 k), and visit schedules, are identical to those of the acupuncture group to maintain procedural consistency.

### Cointerventions

All participants will be required to discontinue pharmacological interventions for KOA throughout the study duration, including both oral and topical agents. Paracetamol will be permitted as a rescue medication following physician-confirmed exacerbations of KOA symptoms, with mandatory documentation of dosage, frequency, and administration timing. Concomitant medications for non-KOA comorbidities may be continued without restrictions. Both groups will be prohibited from receiving adjunctive physical therapies or alternative treatments (eg, massage, moxibustion, or ultrasound therapy). Normal daily activities will be encouraged, while strenuous exercise is discouraged to minimize confounding therapeutic effects.

### Extended Follow-Up

Following the 8-week treatment period, participants will enter a 24-week posttreatment follow-up phase without active interventions. Rescue medication will be permitted in cases of disease progression accompanied by intolerable pain, with paracetamol designated as the primary rescue agent. The daily dosage will be titrated based on symptom severity while ensuring adherence to the maximum daily limit specified in clinical guidelines. If pharmacological intervention fails to alleviate symptoms and surgical indications are met, patients will be referred for surgical evaluation. All rescue interventions, including timing, dosage, frequency, and rationale, will be systematically documented and incorporated into the per-protocol analysis.

### Outcomes

#### Primary Efficacy Outcomes

The primary outcome will be the proportion of responders achieving a clinically meaningful reduction in knee pain at weeks 8 and 24. Response is defined as a 2-point reduction or more from baseline on the Numerical Rating Scale (NRS), where pain intensity ranges from 0 (no pain) to 10 (worst pain). This threshold represents the minimal clinically important difference for the NRS [32]. The NRS, endorsed as the gold standard for pain assessment by the American Pain Society, requires participants to select a single integer corresponding to their current pain intensity using a visual analog scale, where higher scores indicate greater pain severity.

#### Secondary Efficacy Outcomes

The validated instruments mentioned subsequently will assess secondary outcomes.

The first instrument is the Western Ontario and McMaster Universities Osteoarthritis Index (WOMAC). Aligned with the clinical practice guidelines for KOA assessment [43], the WOMAC assesses 5 domains: pain, stiffness, physical function, social participation, and quality of life. The 24-item scale generates a total score ranging from 0 to 240, with higher scores indicating greater disease severity.

The second instrument is the 40 m Fast-Paced Walk Test. This test quantifies knee joint function, lower-limb muscle strength, and ambulatory capacity [44]. Participants complete a 40 m walk at the maximum safe speed; the time (seconds) is recorded by a stopwatch. Shorter durations reflect better functional performance.

The third instrument is the Roland-Morris Disability Questionnaire (RMDQ). RMDQ scores (range 0-24) assess compensatory low back dysfunction secondary to chronic knee pain [45]. Its sensitivity to activity-related impairments captures biomechanical loading alterations and postural adaptations (eg, lumbar muscle overcompensation during gait). Higher scores indicate greater functional limitations.

Fourth, we will assess the depression status. Depressive symptoms will be assessed using the Hamilton Depression Rating Scale-17 [46]. The scale consists of 17 items, with a higher score indicating greater severity of depression.

Fifth, we will assess the anxiety status. Anxiety symptoms will be assessed using the Hamilton Anxiety Rating Scale [47]. The scale comprises 14 items, with a higher score indicating greater severity of anxiety.

Sixth, we will assess the insomnia status. Sleep quality will be assessed using the Insomnia Severity Index (ISI) score [48]. ISI assesses self-reported insomnia symptoms experienced over the past 2 weeks. It consists of 7 items, yielding a total score ranging from 0 to 28, where higher scores indicate more severe insomnia. Notably, it may be challenging to distinguish between insomnia directly related to KOA pain and primary insomnia with items 1 to 3 of the ISI (difficulty falling asleep, difficulty maintaining sleep, and early morning awakenings). To increase the specificity of assessing KOA-related sleep disturbances, we will annotate these items with “caused by KOA.”

We will also assess the pain self-efficacy. The impact of pain on daily functioning will be assessed using the Pain Self-Efficacy Questionnaire (PSEQ) score [49]. PSEQ measures an individual’s confidence in performing activities while experiencing pain. It consists of 10 items, generating a total score ranging from 0 to 60, with higher scores indicating greater self-efficacy beliefs in managing pain-related challenges.

#### Safety Outcomes

Safety assessments will focus on monitoring the incidence of AEs, serious AEs, and acupuncture-related AEs throughout the trial. Acupuncturists are responsible for documenting all AEs, whether deemed treatment related or incidental. Reportable nonserious AEs encompass (1) acupuncture-related incidents, such as hematoma, needling syncope, needle breakage, or retained needles; (2) patient-reported symptoms impacting daily activities; and (3) any laboratory abnormalities exceeding twice the upper limit of normal or falling below half the lower limit of normal. The sham acupuncture group does not carry these specific risks. Standard management protocols for anticipated acupuncture-related AEs are detailed subsequently. First AE includes bruising and hematoma, in which small, localized hematomas will be disinfected and allowed to resolve spontaneously. Symptomatic treatment will be administered by a physician for larger hematomas or instances of significant bleeding. Second AE includes needling syncope, in which acupuncture treatment will be immediately discontinued. Symptomatic management will be provided. Third AE includes needle breakage and retention, in which acupuncture treatment will be discontinued. The affected needle fragment will be addressed (eg, removed, if feasible), and symptomatic management will be provided, as necessary.

In cases of serious AEs (defined as events resulting in hospitalization, persistent or significant disability, or immediate life-threatening conditions), trial participation will be immediately discontinued for the affected participant. The participants will receive comprehensive therapeutic interventions and nursing care at the study site until symptom resolution.

All costs associated with managing intervention-related AEs will be covered by research funding. Detailed documentation of all AEs, including onset time, severity, duration, outcome, and assessed causal relationship to the intervention, will be submitted to the medical ethics committee within 24 hours of occurrence.

### Assessment Schedule

Outcome assessments will be performed at baseline and 4, 8, 16, and 24 weeks after randomization. At the baseline visit, data collection will include patient demographic information (eg, age and sex), anthropometric measurements (height and weight), lifestyle factors, educational attainment, and concomitant treatments. All primary and secondary outcomes and treatment adherence will be evaluated at every scheduled visit. The detailed visit procedures are outlined in Table 2. Any deviations from the study protocol will be recorded. While protocol deviations do not mandate trial withdrawal, they will be accounted for in the per-protocol analysis.

**Table 2:** Study procedures and outcome measures by visit timeline.

Study procedures and outcome measures			Screening		Allocation		Active intervention				Postintervention follow-up		
			Week 0		Week 0		Week 4		Week 8		Week 16		Week 24
**Procedures**													
	Eligibility screening	✓											
	Informed consent from	✓											
	Randomization			✓									
	Acupuncture or sham acupuncture					✓		✓					
**Outcome assessment**													
	Numerical Rating Scale	✓				✓		✓		✓		✓	
	Western Ontario and McMaster Universities Osteoarthritis Index	✓				✓		✓		✓		✓	
	40 m Fast-Paced Walk Test	✓				✓		✓		✓		✓	
	Roland-Morris Disability Questionnaire	✓				✓		✓		✓		✓	
	Hamilton Depression Rating Scale-17	✓				✓		✓		✓		✓	
	Hamilton Anxiety Rating Scale					✓		✓		✓		✓	
	Insomnia Severity Index	✓				✓		✓		✓		✓	
	Pain Self-Efficacy Questionnaire	✓				✓		✓		✓		✓	
	Analgesic use record	✓				✓		✓		✓		✓	
	Patient compliance					✓		✓		✓		✓	
	Blind evaluation					✓		✓					
	Adverse event record					✓		✓		✓		✓	

### Quality Assurance

Case report forms for each participant will be archived in individual paper-based folders in secure, access-controlled storage to ensure confidentiality. Data from paper case report forms will undergo dual independent entry by 2 trained researchers into a designated electronic database. All discrepancies will be resolved through source data verification conducted by a third-party auditor, referencing original medical records. All personnel handling the study data will sign legally binding confidentiality agreements compliant with international regulations.

An independent data and safety monitoring board (DSMB), comprising multidisciplinary experts free of conflicts of interest, will be established to safeguard participant welfare and ensure trial integrity across implementation, monitoring, and data interpretation. The DSMB’s responsibilities include overseeing interim analyses and determining trial continuation based on prespecified evaluations of safety profiles, efficacy signals, and protocol adherence rates. DSMB will conduct monthly reviews of safety data, including the frequency of all nonserious AEs and serious AEs, along with causal attribution assessments to the trial intervention. To address ethical considerations regarding the sham acupuncture group not receiving therapeutic acupuncture during the trial, all participants in this group will receive a complimentary equivalent course of acupuncture upon trial completion. This approach upholds the principle of distributive justice while maintaining enrollment retention and protocol compliance.

### Data Management and Sharing

We will implement a rigorous data protection framework to ensure patient privacy and data security. During data collection, only participant IDs and anonymized aliases will be recorded, avoiding sensitive identifiers, such as national ID numbers. All physical documents will be stored in encrypted cabinets, while electronic data will be transmitted exclusively through secure channels. The database will enforce tiered access privileges with comprehensive activity logging. For multicenter collaboration, all research sites will transmit data via encrypted virtual private network tunnels and adhere to unified collection protocols. Upon trial completion, all datasets will undergo deidentification before storage on dedicated servers. Any future data sharing requires prior approval by an independent ethics committee. The entire data management process strictly complies with China’s Personal Information Protection Law requirements. All researchers must sign confidentiality agreements and will be subject to continuous oversight by the DSMB.

### Sample Size Calculation

We hypothesize that acupuncture is more effective than sham acupuncture in alleviating symptoms of KOA. On the basis of our pilot study data, we conservatively estimate response rates (the primary outcome) of 82% for the acupuncture group versus 65% for the sham acupuncture group. With a 2-sided α level of .05, a statistical power of 90%, and an allowable attrition rate of 20%, the sample size calculation resulted in a requirement of 343 participants per group. To enhance statistical robustness, we expanded the sample size to 350 cases, with 175 (50%) cases in both groups.

### Statistical Analysis Plan

Data analysis will be conducted using SAS (version 9.4; SAS Institute Inc). In baseline analyses, continuous variables conforming to a normal distribution will be presented as means and SDs, whereas nonnormally distributed continuous variables will be summarized as medians with IQRs. Categorical variables will be expressed as frequencies and percentages. Normality will be assessed using the Kolmogorov-Smirnov test. For between-group comparisons of normally distributed outcome data with homogeneity of variance (verified by the Levene test), independent-sample 2-tailed *t* tests will be used. Nonnormally distributed outcome data will be analyzed using the Mann-Whitney *U* test. Differences in categorical variables between groups will be evaluated by the chi-square test.

For primary outcomes, generalized linear mixed models adjusted for prespecified covariates (sex, age, disease duration, baseline NRS score, BMI, occupation, study site, visit time, and group-time interaction) will be used to estimate treatment effects, with study sites modeled as random effects. To address multiplicity, significance testing for primary outcomes will follow a sequential testing approach: only if week 8 shows between-group significance can week 24 results be deemed significant. The tests for secondary outcomes will not be corrected for multiplicity because all secondary outcomes are for exploratory intentions. All analyses will follow the modified intention-to-treat principle, including all participants who received at least 1 session of the trial intervention and provided at least 1 postbaseline NRS score. To validate the missing at random assumption for missing data, we will implement a logistic regression model where the missingness indicator (binary status: missing vs observed) serves as the dependent variable, with treatment group allocation and baseline characteristics as covariates. The missing at random assumption is supported if the model demonstrates statistical significance (eg, treatment group affecting missing probability) without including the outcome variables themselves [50]. Missing data at random will be imputed using multiple imputation methods; categorical variables will be imputed via logistic regression models, and continuous variables will be imputed via linear regression models. The covariates included in the imputation model are identical to the confounding factors adjusted for in the primary analysis model, including sex, age, disease duration, baseline NRS score, BMI, occupation, study site, visit time, and group-time interaction. Data missing not at random will not be imputed. All the statistical tests will use a 2-sided α level of .05, with the results reported using 95% CIs.

To evaluate the robustness of effect estimates, two sensitivity analyses will be conducted: (1) a per-protocol analysis restricted to participants who completed both week 8 and week 24 visits, attended 80% or more acupuncture or sham acupuncture sessions, maintained blinding status, and refrained from all prohibited interventions (including rescue therapies) and (2) a complete-case analysis without missing data imputation.

Blinding success will be evaluated using the James and Bang indices [51]. The James index (range 0-1) measures overall blinding effectiveness based on “don’t know” responses, where higher values indicate better blinding. The Bang index (range –1 to 1) separately analyzes correct guessing rates for each group, where positive values suggest blinding failure, negative values represent successful blinding, and the absolute magnitude reveals intergroup differences.

## Results

This study received funding in November 2024. Participant recruitment has been initiated. The first participant was enrolled on February 15, 2025. As of June 28, 2025, we have recruited 25 participants. Data analysis has not commenced. The final data collection is expected to be completed by March 2026, and the anticipated publication date for the study results is December 2027.

## Discussion

### Overview

KOA is recognized as one of the most prevalent chronic joint disorders in middle-aged and older adult populations. This condition imposes dual burdens through progressive functional disability that substantially compromises quality of life and escalates health care expenditures that strain medical systems. Although key pathological mechanisms, including articular cartilage degeneration, osteophyte formation, synovial inflammation, and altered synovial fluid composition, are well characterized [52], the therapeutic management of KOA remains suboptimal due to the persistent absence of clinically effective, mechanistically targeted disease-modifying therapies.

Although multiple studies have reported positive effects of acupuncture in KOA management, methodological limitations, such as small sample sizes, suboptimal study designs, and short-term follow-up durations, have precluded definitive conclusions [38,53]. A meta-analysis of acupuncture for KOA suggested superior efficacy over sham acupuncture or usual care in pain relief and functional improvement, yet it yielded low-certainty evidence due to the prevalent high risk of bias among the included trials [54]. Another meta-analysis incorporating 10 RCTs demonstrated significant acupuncture-induced improvements in pain and functional status among patients with KOA; however, considerable heterogeneity among these RCTs compromised the reliability of the results [55]. Furthermore, insufficient standardization of acupuncture protocols compromises comparability and reproducibility across the RCTs [56]. A methodological systematic review highlighted recurrent deficiencies in descriptions of acupuncture interventions, acupoint selection rationales, and acupuncturist qualifications, thereby undermining evidence credibility [57]. These limitations underscore the imperative of large-scale, multicenter RCTs with standardized protocols for the objective evaluation of acupuncture efficacy and safety profiles in patients with KOA. Notably, the clinical implications of acupoint sensitization phenomena, recently identified as potential therapeutic enhancers, warrant further validation through clinical trials.

### Innovations and Limitations

Given these considerations, we designed this multicenter RCT across 3 clinical sites to evaluate the efficacy and safety of sensitization-guided acupoint selection in acupuncture for treating KOA. This study incorporates 3 methodological advantages. First, the use of Takakura sham acupuncture devices enables simulated needle insertion, generating cutaneous perception of penetration without actual skin puncture, thereby achieving robust participant blinding. This rigorous blinding protocol ensures that observed intergroup differences arise from biological mechanisms rather than expectancy effects or procedural psychological influences, substantially enhancing scientific validity. Second, our sensitization-based acupoint selection strategy potentially enhances therapeutic responses. Third, the multicenter design with sufficient statistical power ensures the generalizability of the results while minimizing selection bias through centralized randomization and standardized protocols. Participants, outcome assessors, and data analysts maintain blinding throughout the trial to prevent performance, detection, and reporting bias. Therefore, we believe that this RCT will generate high-quality evidence to inform clinical practice.

This RCT has several potential limitations. First, blinding of acupuncturists is inherently unfeasible. Given their awareness of group allocation and responsibility for intervention delivery, acupuncturists may inadvertently or intentionally exert undue influence on participants, such as administering additional acupuncture sessions to the treatment group or reducing the proactive provision of lifestyle advice to the control group. These factors may introduce performance bias that could overestimate the therapeutic effects of acupuncture. To mitigate such bias, we implemented standardized operating procedures, restricted nontherapeutic clinician-participant communication, and established on-site monitoring protocols to ensure protocol adherence, as detailed in the Blinding Procedures section. Second, the selection of the NRS as the primary outcome may be susceptible to subjectivity. Although randomization balances subjective effects across groups, residual bias may persist. To address this, we established a multidimensional secondary outcome system to triangulate NRS results, including composite scale assessments (ie, WOMAC, RMDQ, Hamilton Depression Rating Scale-17, Hamilton Anxiety Rating Scale, ISI, and PSEQ) and objectively measurable end points (40 m Fast-Paced Walk Test). In addition, biological markers (eg, interleukin-1β and tumor necrosis factor-α) are excluded due to insufficient evidence correlating inflammatory factors with KOA pain severity or disease progression. Third, despite efforts to minimize placebo effects (eg, using nonpenetrating sham needle devices), residual placebo responses may introduce bias. Crucially, such bias would likely attenuate detectable differences between true and sham acupuncture groups. If statistically significant differences persist despite this conservative bias, they would provide stronger evidence for acupuncture’s efficacy, aligning with the objective of this trial.

### Communication Plan for Major Protocol Amendments

Any protocol amendments occurring after trial initiation, including adjustments to eligibility criteria, refinements in efficacy evaluation methods, or changes in outcome measures, will require formal approval by unanimous agreement among all relevant stakeholders (eg, the institutional review board and enrolled participants), complying with predefined communication procedures. Modifications directly impacting participants will be disseminated immediately through multichannel communication systems (eg, updated informed consent forms and specialized helplines), supplemented by structured interviews to actively solicit participant feedback. A standardized survey mechanism will be deployed continuously during the trial to quantitatively assess participant satisfaction with amendment processes, thereby guaranteeing auditable transparency practices and robust protection of participant rights through institutional grievance resolution frameworks.

### Conclusions

In summary, this RCT aims to establish a protocol for applying acupuncture based on acupoint sensitization as a therapeutic approach to KOA management. If the anticipated therapeutic efficacy is demonstrated, this acupuncture approach may serve as a preferred therapeutic alternative for KOA management, aiming to reduce pharmacological dependency, mitigate adverse drug reactions, and ultimately enhance the quality of life for affected populations. Furthermore, the findings of this RCT can be expected to contribute to the development of clinical guidelines for KOA treatment and advance the evidence-based standardization of acupuncture protocols within KOA management.

## Appendix

Multimedia Appendix 1 SPIRIT checklist.

Multimedia Appendix 2 Standardized training records and consistency assessment protocol.

## Abbreviations

AE: adverse event
DSMB: data and safety monitoring board
ISI: Insomnia Severity Index
KOA: knee osteoarthritis
NRS: Numerical Rating Scale
PSEQ: Pain Self-Efficacy Questionnaire
RCT: randomized controlled trial
RMDQ: Roland-Morris Disability Questionnaire
SPIRIT: Standard Protocol Items: Recommendations for Interventional Trials
TKA: total knee arthroplasty
WOMAC: Western Ontario and McMaster Universities Osteoarthritis Index

## References

[1] Tang S, Zhang C, Oo WM, Fu K, Risberg MA, Bierma-Zeinstra SM, Neogi T, Atukorala I, Malfait AM, Ding C, Hunter DJ (2025). Osteoarthritis. Nat Rev Dis Primers.

[2] Cross M, Smith E, Hoy D, Nolte S, Ackerman I, Fransen M, Bridgett L, Williams S, Guillemin F, Hill CL, Laslett LL, Jones G, Cicuttini F, Osborne R, Vos T, Buchbinder R, Woolf A, March L (2014). The global burden of hip and knee osteoarthritis: estimates from the global burden of disease 2010 study. Ann Rheum Dis.

[3] Neogi T, Zhang Y (2013). Epidemiology of osteoarthritis. Rheum Dis Clin North Am.

[4] Lespasio MJ, Piuzzi NS, Husni ME, Muschler GF, Guarino A, Mont MA (2017). Knee osteoarthritis: a primer. Perm J.

[5] Losina E, Katz JN (2012). Total knee arthroplasty on the rise in younger patients: are we sure that past performance will guarantee future success?. Arthritis Rheum.

[6] Losina E, Paltiel AD, Weinstein AM, Yelin E, Hunter DJ, Chen SP, Klara K, Suter LG, Solomon DH, Burbine SA, Walensky RP, Katz JN (2015). Lifetime medical costs of knee osteoarthritis management in the United States: impact of extending indications for total knee arthroplasty. Arthritis Care Res (Hoboken).

[7] Jin X, Liang W, Zhang L, Cao S, Yang L, Xie F (2023). Economic and humanistic burden of osteoarthritis: an updated systematic review of large sample studies. Pharmacoeconomics.

[8] Larsson SC, Burgess S (2022). Appraising the causal role of smoking in multiple diseases: a systematic review and meta-analysis of Mendelian randomization studies. EBioMedicine.

[9] Veronese N, Stubbs B, Solmi M, Smith TO, Noale M, Cooper C, Maggi S (2017). Association between lower limb osteoarthritis and incidence of depressive symptoms: data from the osteoarthritis initiative. Age Ageing.

[10] Kolasinski SL, Neogi T, Hochberg MC, Oatis C, Guyatt G, Block J, Callahan L, Copenhaver C, Dodge C, Felson D, Gellar K, Harvey WF, Hawker G, Herzig E, Kwoh CK, Nelson AE, Samuels J, Scanzello C, White D, Wise B, Altman RD, DiRenzo D, Fontanarosa J, Giradi G, Ishimori M, Misra D, Shah AA, Shmagel AK, Thoma LM, Turgunbaev M, Turner AS, Reston J (2020). 2019 American College of Rheumatology/Arthritis Foundation guideline for the management of osteoarthritis of the hand, hip, and knee. Arthritis Rheumatol.

[11] Bhala N, Emberson J, Merhi A, Abramson S, Arber N, Baron JA, Bombardier C, Cannon C, Farkouh ME, FitzGerald GA, Goss P, Halls H, Hawk E, Hawkey C, Hennekens C, Hochberg M, Holland LE, Kearney PM, Laine L, Lanas A, Lance P, Laupacis A, Oates J, Patrono C, Schnitzer TJ, Solomon S, Tugwell P, Wilson K, Wittes J, Baigent C, Coxib and traditional NSAID Trialists' (CNT) Collaboration (2013). Vascular and upper gastrointestinal effects of non-steroidal anti-inflammatory drugs: meta-analyses of individual participant data from randomised trials. Lancet.

[12] Zhang W, Moskowitz RW, Nuki G, Abramson S, Altman RD, Arden N, Bierma-Zeinstra S, Brandt KD, Croft P, Doherty M, Dougados M, Hochberg M, Hunter DJ, Kwoh K, Lohmander LS, Tugwell P (2008). OARSI recommendations for the management of hip and knee osteoarthritis, Part II: OARSI evidence-based, expert consensus guidelines. Osteoarthritis Cartilage.

[13] Dowell D, Haegerich TM, Chou R (2016). CDC guideline for prescribing opioids for chronic pain--United States, 2016. JAMA.

[14] Krebs EE, Gravely A, Nugent S, Jensen AC, DeRonne B, Goldsmith ES, Kroenke K, Bair MJ, Noorbaloochi S (2018). Effect of opioid vs nonopioid medications on pain-related function in patients with chronic back pain or hip or knee osteoarthritis pain: the SPACE randomized clinical trial. JAMA.

[15] Fransen M, McConnell S, Harmer AR, Van der Esch M, Simic M, Bennell KL (2015). Exercise for osteoarthritis of the knee: a Cochrane systematic review. Br J Sports Med.

[16] Franco MR, Morelhão PK, de Carvalho A, Pinto RZ (2017). Aquatic exercise for the treatment of hip and knee osteoarthritis. Phys Ther.

[17] Bennell KL, Dobson F, Hinman RS (2014). Exercise in osteoarthritis: moving from prescription to adherence. Best Pract Res Clin Rheumatol.

[18] Skou ST, Roos EM, Laursen MB, Rathleff MS, Arendt-Nielsen L, Simonsen O, Rasmussen S (2015). A randomized, controlled trial of total knee replacement. N Engl J Med.

[19] Wylde V, Hewlett S, Learmonth ID, Dieppe P (2011). Persistent pain after joint replacement: prevalence, sensory qualities, and postoperative determinants. Pain.

[20] Bayliss LE, Culliford D, Monk AP, Glyn-Jones S, Prieto-Alhambra D, Judge A, Cooper C, Carr AJ, Arden NK, Beard DJ, Price AJ (2017). The effect of patient age at intervention on risk of implant revision after total replacement of the hip or knee: a population-based cohort study. Lancet.

[21] Vickers AJ, Vertosick EA, Lewith G, MacPherson H, Foster NE, Sherman KJ, Irnich D, Witt CM, Linde K (2018). Acupuncture for chronic pain: update of an individual patient data meta-analysis. J Pain.

[22] Zhang R, Lao L, Ren K, Berman BM (2014). Mechanisms of acupuncture-electroacupuncture on persistent pain. Anesthesiology.

[23] Lin LL, Li YT, Tu JF, Yang JW, Sun N, Zhang S, Wang TQ, Shi GX, Du Y, Zhao JJ, Xiong DC, Hou HK, Liu CZ (2018). Effectiveness and feasibility of acupuncture for knee osteoarthritis: a pilot randomized controlled trial. Clin Rehabil.

[24] Mei F, Yao M, Wang Y, Ma Y, Liu Y, Wu M, Wang Z, Feng L, Hu K, Ma B (2023). Acupuncture for knee osteoarthritis: a systematic review and meta-analysis. J Evid Based Med.

[25] Sun J, Liang Y, Luo KT, Shao XM, Tu MQ, Wu XT, Liu F, Li XW, Chen YD, Zhang QF, Ji CH, Li RR, Li XY, Xu F, Fang JQ (2025). Efficacy of different acupuncture techniques for pain and dysfunction in patients with knee osteoarthritis: a randomized controlled trial. Pain Ther.

[26] Wang TQ, Li LR, Tan CX, Yang JW, Shi GX, Wang LQ, Hu H, Liu ZS, Wang J, Wang T, Yuan Y, Jia WR, Li H, Wang XW, Wu B, Tu JF, Liu CZ (2021). Effect of electroacupuncture on gut microbiota in participants with knee osteoarthritis. Front Cell Infect Microbiol.

[27] Liu XY, Ma Y, Huang ZY, Xiao XX, Guan L (2024). The efficacy of acupuncture, exercise rehabilitation, and their combination in the treatment of knee osteoarthritis: a randomized controlled trial. J Pain Res.

[28] Witt C, Brinkhaus B, Jena S, Linde K, Streng A, Wagenpfeil S, Hummelsberger J, Walther HU, Melchart D, Willich SN (2005). Acupuncture in patients with osteoarthritis of the knee: a randomised trial. Lancet.

[29] Tu JF, Yang JW, Shi GX, Yu ZS, Li JL, Lin LL, Du YZ, Yu XG, Hu H, Liu ZS, Jia CS, Wang LQ, Zhao JJ, Wang J, Wang TQ, Wang Y, Wang T, Zhang N, Zou X, Wang Y, Shao JK, Liu CZ (2021). Efficacy of intensive acupuncture versus sham acupuncture in knee osteoarthritis: a randomized controlled trial. Arthritis Rheumatol.

[30] Ng HP, Tan CY, Lim CJ, Tan TL, Yang S, Long GS, Tan SI, Chua YC, Yan YW, Soh DB, Goh TH, Ng PJ, Ng YT, Kuan SB, Teo BS, Kong KH, Ho G, Koh HQ, Pereira MJ, Tan BY (2025). Heat and acupuncture restore mobility in knee osteoarthritis (HARMOKnee): a pragmatic integrated care, randomized controlled study. Complement Ther Med.

[31] Bellamy N, Buchanan WW, Goldsmith CH, Campbell J, Stitt LW (1988). Validation study of WOMAC: a health status instrument for measuring clinically important patient relevant outcomes to antirheumatic drug therapy in patients with osteoarthritis of the hip or knee. J Rheumatol.

[32] Hawker GA, Mian S, Kendzerska T, French M (2011). Measures of adult pain: Visual Analog Scale for Pain (VAS Pain), Numeric Rating Scale for Pain (NRS Pain), McGill Pain Questionnaire (MPQ), Short-Form McGill Pain Questionnaire (SF-MPQ), Chronic Pain Grade Scale (CPGS), Short Form-36 Bodily Pain Scale (SF-36 BPS), and Measure of Intermittent and Constant Osteoarthritis Pain (ICOAP). Arthritis Care Res (Hoboken).

[33] Sun N, Wang LQ, Shao JK, Zhang N, Zhou P, Fang SN, Chen W, Yang JW, Liu CZ (2020). An expert consensus to standardize acupuncture treatment for knee osteoarthritis. Acupunct Med.

[34] Tu JF, Wang XZ, Yan SY, Wang YR, Yang JW, Shi GX, Zhang WZ, Jin LN, Yang LS, Liu DH, Wang LQ, Mi BH (2025). Thermal sensitization of acupoints in patients with knee osteoarthritis: a cross-sectional case-control study. J Integr Med.

[35] Luo X, Liu J, Li Q, Zhao J, Hao Q, Zhao L, Chen Y, Yin P, Li L, Liang F, Sun X (2023). Acupuncture for treatment of knee osteoarthritis: a clinical practice guideline. J Evid Based Med.

[36] Chan AW, Tetzlaff JM, Gøtzsche PC, Altman DG, Mann H, Berlin JA, Dickersin K, Hróbjartsson A, Schulz KF, Parulekar WR, Krleza-Jeric K, Laupacis A, Moher D (2013). SPIRIT 2013 explanation and elaboration: guidance for protocols of clinical trials. BMJ.

[37] Hochberg MC, Altman RD, Brandt KD, Clark BM, Dieppe PA, Griffin MR, Moskowitz RW, Schnitzer TJ (1995). Guidelines for the medical management of osteoarthritis. Part II. Osteoarthritis of the knee. American College of Rheumatology. Arthritis Rheum.

[38] MacPherson H, Vertosick EA, Foster NE, Lewith G, Linde K, Sherman KJ, Witt CM, Vickers AJ (2017). The persistence of the effects of acupuncture after a course of treatment: a meta-analysis of patients with chronic pain. Pain.

[39] Xu GX, Zhou YM, Sun MS, Luo LJ, Liu XJ, Wang D, Zhao L, Cai DJ, Chen J, Zheng H, Ji LX, Cui J, Chang XR, Liang FR (2020). [Clinical observation on distribution characteristics and rules of pain sensitivity points on body surface in patients with knee osteoarthritis]. Zhongguo Zhen Jiu.

[40] Wang RH, Xue PJ, Xing HJ, Jia CS, Shi J (2022). [Complex network analysis on regularities of acupoint combinations and application characteristics of acupuncture and moxibustion in the treatment of knee osteoarthritis]. Zhen Ci Yan Jiu.

[41] Lim S (2010). WHO standard acupuncture point locations. Evid Based Complement Alternat Med.

[42] 3Dbody homepage. 3Dbody.

[43] Melzack R (1987). The short-form McGill Pain Questionnaire. Pain.

[44] Allen KD, Woolson S, Hoenig HM, Bongiorni D, Byrd J, Caves K, Hall KS, Heiderscheit B, Hodges NJ, Huffman KM, Morey MC, Ramasunder S, Severson H, Van Houtven C, Abbate LM, Coffman CJ (2021). Stepped exercise program for patients with knee osteoarthritis : a randomized controlled trial. Ann Intern Med.

[45] Edelen MO, Rodriguez A, Herman P, Hays RD (2021). Crosswalking the patient-reported outcomes measurement information system physical function, pain interference, and pain intensity scores to the Roland-Morris Disability Questionnaire and the Oswestry Disability Index. Arch Phys Med Rehabil.

[46] Kølbæk P, Nielsen CW, Buus CW, Friis SR, Nilsson E, Jensen BD, Bueno AV, Østergaard SD (2024). Clinical validation of the self-reported 6-item Hamilton Depression Rating Scale (HAM-D6-SR) among inpatients. J Affect Disord.

[47] Zimmerman M, Martin J, Clark H, McGonigal P, Harris L, Holst CG (2017). Measuring anxiety in depressed patients: a comparison of the Hamilton anxiety rating scale and the DSM-5 Anxious Distress Specifier Interview. J Psychiatr Res.

[48] Badahdah AM, Khamis F, Aloud N (2025). Evaluation of a brief three-item Insomnia Severity Index (ISI-3) among healthcare workers. Behav Sleep Med.

[49] Dubé MO, Langevin P, Roy JS (2021). Measurement properties of the Pain Self-Efficacy Questionnaire in populations with musculoskeletal disorders: a systematic review. Pain Rep.

[50] van Buuren S (2018). Flexible Imputation of Missing Data, Second Edition.

[51] Bang H, Ni L, Davis CE (2004). Assessment of blinding in clinical trials. Control Clin Trials.

[52] Primorac D, Molnar V, Rod E, Jeleč Ž, Čukelj F, Matišić V, Vrdoljak T, Hudetz D, Hajsok H, Borić I (2020). Knee osteoarthritis: a review of pathogenesis and state-of-the-art non-operative therapeutic considerations. Genes (Basel).

[53] Chen N, Wang J, Mucelli A, Zhang X, Wang C (2017). Electro-acupuncture is beneficial for knee osteoarthritis: the evidence from meta-analysis of randomized controlled trials. Am J Chin Med.

[54] Manheimer E, Cheng K, Linde K, Lao L, Yoo J, Wieland S, van der Windt DA, Berman BM, Bouter LM (2010). Acupuncture for peripheral joint osteoarthritis. Cochrane Database Syst Rev.

[55] Chen H, Shi H, Gao S, Fang J, Yi J, Wu W, Liu X, Liu Z (2024). Durable effects of acupuncture for knee osteoarthritis: a systematic review and meta-analysis. Curr Pain Headache Rep.

[56] MacPherson H, Altman DG, Hammerschlag R, Youping L, Taixiang W, White A, Moher D (2010). Revised STandards for Reporting Interventions in Clinical Trials of Acupuncture (STRICTA): extending the CONSORT statement. J Altern Complement Med.

[57] White AR, Ernst E (1999). A systematic review of randomized controlled trials of acupuncture for neck pain. Rheumatology (Oxford).

