# Supplementary material for: Efficacy and Safety of Acupuncture at Sensitized Acupoints for Knee Osteoarthritis: Protocol for a Multicenter, Single-Blind Randomized Controlled Trial
Source: JMIR Res Protoc. 2025 Sep 24;14:e77336. doi: 10.2196/77336 (PMC12508665; doi:10.2196/77336)
Supplement: Multimedia Appendix 2 [file resprot_v14i1e77336_app2.pdf]

Supplementary file 2

Standardized Training Records and Consistency Assessment Protocol

1. Acupoint Localization

The primary acupoints selected are Chize (LU5), Quchi (LI11), Dubi (ST35), Fengshi (GB31), and Xiyangguan (GB33) on the affected side (Figure 1). Localization adheres to the WHO Standard Acupuncture Point Location in the Western Pacific Region:

- LU5 (Chize): On the anterior cubital crease, radial to the tendon of the biceps brachii muscle.
- LI11 (Quchi): On the lateral aspect of the elbow, at the midpoint of the line connecting the lateral end of the cubital crease and the lateral epicondyle of the humerus, measured with the elbow in flexion.
- ST35 (Dubi): On the anterolateral aspect of the knee, within the lateral depression of the patellar ligament.
- GB31 (Fengshi): On the lateral midline of the thigh, 20 cm proximal to the popliteal crease, in the depression midway between the greater trochanter and the lateral femoral epicondyle (located between the vastus lateralis and the biceps femoris muscles).
- GB33 (Xiyangguan): On the lateral aspect of the knee, in the depression immediately superior to the lateral epicondyle of the femur.

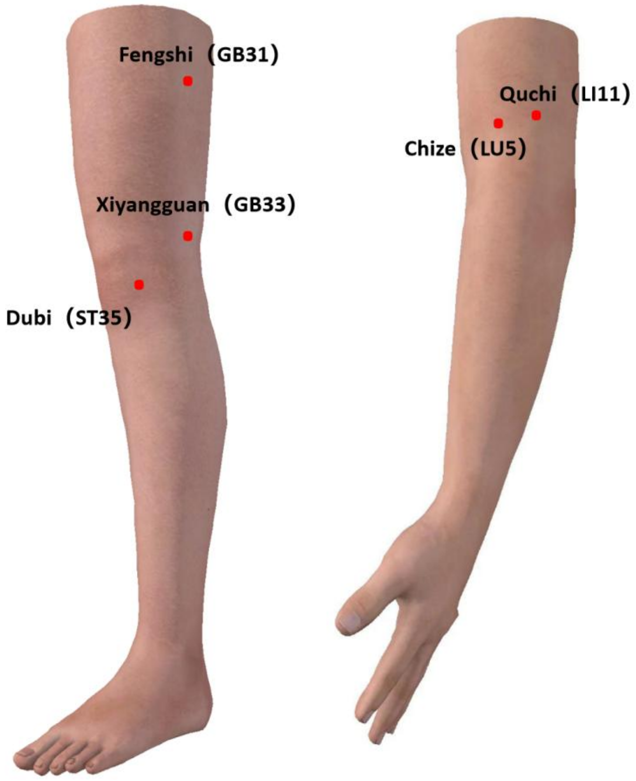

Figure 1. Anatomical location of selected acupoints (source: www.3Dbody.com)

2. Environment

Maintain a quiet and well-lit indoor environment. Soft background music may be used appropriately. The consultation room temperature should be maintained at approximately 22°C to 26°C.

3. Patient Position

Instruct the patient to adopt a comfortable position that fully exposes the area to be examined. The patient should relax their muscles, breathe evenly, concentrate their mind, and focus on the sensations experienced during the examination.

4. Standard Operating Procedure

(1) Acupuncture Group

- **Instrumentation:** Sterile disposable Huatuo-brand acupuncture needles (diameter 0.30 mm × length 40 mm).
- **Procedure:**

- Disinfection: Disinfect the skin over the acupoint using a 75% ethanol swab in a circular motion (diameter ≥ 3 cm).
- Needle Insertion: Quickly penetrate the skin and then insert the needle slowly and perpendicularly. Adjust the needle body until the patient reports no sharp pain.
- Needle Manipulation: Apply a combination of lifting-thrusting and rotating methods for 30 seconds per acupoint (lifting-thrusting amplitude 3-5 mm, rotating angle 180°-360°, frequency 2 Hz). The intensity should be sufficient to elicit de qi sensations (characterized by soreness, numbness, distension, or heaviness) and remain tolerable to the patient.
- Needle Retention: Retain needles for 30 minutes, with repeated manual stimulation performed every 10 minutes. After needle removal, apply pressure to the insertion site with a sterile cotton ball for 30 seconds. Detailed specifications for each acupoint are provided in Table 1.

Table 1. Details of Acupoint Manipulation

**Acupoint**	**Details**
LU5 (Chize)	- Depth: 25-30 mm (reaching the brachioradialis muscle layer)<br>- Direction: Perpendicular to the skin<br>- Caution: Avoid radial artery (confirm absence of pulse by palpation)
LI11 (Quchi)	- Depth: 25-30 mm (reaching the extensor carpi radialis longus muscle layer)<br>- Direction: Perpendicular to the skin<br>- Caution: Avoid excessive manipulation to prevent stimulation of the superficial branch of the radial nerve
ST35 (Dubi)	- Depth: 15-20 mm (superficial insertion to subcutaneous fat layer)<br>- Direction: Needle tip angled slightly towards the center of the patella (15°)<br>- Caution: Do not penetrate deeply into the joint cavity
GB31 (Fengshi)	- Depth: 30-35 mm (reaching the deep layer of the fascia lata)<br>- Direction: Perpendicular to the skin<br>- Caution: Avoid branches of the lateral femoral cutaneous nerve (inquire about radiating pain)
GB33	- Depth: 20-25 mm (reaching the deep layer of the iliotibial tract)

Acupoint	Details
(Xiyangguan)	- Direction: Needle tip angled towards the knee joint cavity (30°)<br>- Caution: Avoid puncturing the knee joint synovial bursa

(2) Sham Acupuncture Group

Instrumentation: Takakura non-penetrating sham devices (Figure 2), comprising a blunted-tip sham needle (visually identical to a verum needle), a retractable sleeve, a double-sided adhesive base, and a fixation ring.

Procedure:

- **Device Fixation:** Affix the double-sided adhesive base to the skin over the acupoint. Insert the blunted-tip sham needle into the sleeve assembly.
- **Simulated Manipulation:** Press the top of the device to retract the sleeve (simulating needle insertion). The sham needle tip only contacts the skin surface (no penetration). Imitate the verum acupuncture manipulation procedure for 30 seconds (device movement trajectory matching lifting-thrusting and rotating).
- **Simulated Retention:** Maintain the device in place for 30 minutes before removal, leaving no wound on the skin.

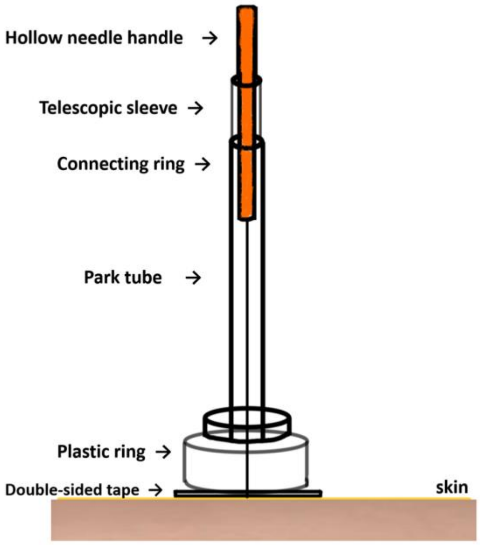

Figure 3. Sham acupuncture device.

5. Treatment Schedule and Quality Control

- **Frequency:** Three sessions per week for 8 consecutive weeks (total 24 sessions).
- **Operator Qualification:** Procedures must be performed by licensed acupuncturists with ≥10 years of clinical experience, all of whom have successfully completed a 2-week protocol-specific standardized training program.
- **Consistency Assurance:** Acupoint localization is verified prior to each treatment session (referencing Figure 1). The intensity of needle manipulation is supervised and recorded by an independent monitor.

6. Strategies to Avoid Performance Bias

During treatment, a clinical monitor escorts the patient to the treatment room. After the patient assumes the supine position, the monitor draws a curtain suspended above the bed to physically separate the patient's head and torso. The monitor then informs the acupuncturist to enter and perform the standardized acupuncture procedure. Throughout the treatment session, the acupuncturist is prohibited from inquiring about any patient information unrelated to the treatment, including contact details. Upon completion of the treatment, the acupuncturist leaves the treatment room first, followed by the patient, minimizing opportunities for contact and communication. The monitor is responsible for supervising the entire treatment process to ensure adherence to the protocol.

7. Consistency Assessment

A simulation test will be conducted to evaluate the standardization of the intervention based on the items listed in Table 2. This assessment will be performed by two senior acupuncturists, each with over 20 years of clinical experience (one of whom is the Principal Investigator of this study). Acupuncturists must pass all items in this assessment before they are qualified to perform treatments in this study.

Table 2. Consistency assessment items and pass criteria.

Consistency Assessment Item	Pass Criteria	Result
Stability of consultation room environmental parameters	Temperature 22-26 ℃ ± 1℃ / Humidity 40-60% ± 5% / Illuminance ≥ 300 lux	Pass □ Fail □
Coincidence of needling location with acupoint location	Error ≤1 mm	Pass □ Fail □
Depth of needle insertion conforms to WHO standard	Conforms to WHO Standard	Pass □ Fail □
Consistency of manipulation parameters (amplitude/angle/frequency)	Conforms to Lifting-Thrusting 3-5 mm / Rotating 180°-360° / Frequency 2 Hz ± 0.2 Hz	Pass □ Fail □
Sham device operation consistency	Sleeve retraction amplitude = Verum needle depth ± 1 mm; Movement trajectory matches lifting-thrusting/rotating	Pass □ Fail □
Needle retention time and interval time	30±2 minutes; Manipulation every 10 minutes (error ≤ 1 minute)	Pass □ Fail □
Elicitation of de qi sensation during manipulation	Patient reports *de qi* sensations (soreness, numbness, distension, heaviness)	Pass □ Fail □
Integrity of needles/devices	Verum needle: No bending/rust; Sham needle: Blunt tip (non-penetrating), smooth sleeve retraction	Pass □ Fail □
Specification of sterile disposable acupuncture needles	Conforms: Huatuo-brand, diameter 0.30 mm × length 40 mm	Pass □ Fail □
Implementation of physical isolation measures	Curtain completely blocks patient's line of sight (visibility between head and torso = 0)	Pass □ Fail □
Adherence to "Strategies to Avoid Performance Bias"	No non-therapeutic communication with patients during treatment or post-treatment sessions	Pass □ Fail □
